# Review of "*Infectious Disease: a Scientific American reader*."

**DOI:** 10.1186/1756-3305-1-36

**Published:** 2008-09-30

**Authors:** Chris Arme

**Affiliations:** 1 School of Life Sciences, Keele University, Staffordshire, ST5 5BG, UK

## Review

This Reader is a collection of some 30 articles on infectious disease, published since 1993. After an essay on *Evolution and the Origins of Disease *there are four sections, each with a common theme.

The first deals with viruses and considers general questions such as *Are viruses alive? *and *Emerging viruses *to more specific topics such as influenza, hepatitis C, HIV and rabies. The second contains something of a mixed bag of *Helicobacter*, STDs, malaria, anthrax and prion diseases. There are seven chapters in the section on the immune system and the final section comprises six essays on more general themes including pandemics, global climate change and antibiotic resistance.

Most of the chapters contain illustrations in black and white and suggestions are offered for further reading, although these might have been more useful if they had been updated to include more recent references. There is no index.

Although the oldest article was first published in 1993 and seven others appeared in the late 1990s, the rest are from the past 8 years, with the latest from 2007. It is probable that experts in the fields covered by the essays will consider them out-of-date. However, as a general reader, I found them to be fresh and full of ideas that caught my interest.

This collection is an enjoyable read and I commend it to colleagues who seek a flavour of topics in infectious disease, about which they may know little and would like to know more.

## Competing interests

The author declares that he has no competing interests.

